# Evidence-based pain management: is the concept of integrative medicine applicable?

**DOI:** 10.1186/1878-5085-3-13

**Published:** 2012-10-22

**Authors:** Rostyslav V Bubnov

**Affiliations:** 1 The Centre of Ultrasound Diagnostics and Interventional Sonography, Clinical Hospital ‘Pheophania’ of State Affairs Department, Zabolotny str., 21, Kyiv, 03680, Ukraine

**Keywords:** Predictive, Preventive, and Personalized medicine, Participating medicine, Myofascial pain, Pain therapy, Acupuncture, Dry needling, Ultrasound, Neurophysiology study

## Abstract

This article is dedicated to the concept of predictive, preventive, and personalized (integrative) medicine beneficial and applicable to advance pain management, overviews recent insights, and discusses novel minimally invasive tools, performed under ultrasound guidance, enhanced by model-guided approach in the field of musculoskeletal pain and neuromuscular diseases. The complexity of pain emergence and regression demands intellectual-, image-guided techniques personally specified to the patient. For personalized approach, the combination of the modalities of ultrasound, EMG, MRI, PET, and SPECT gives new opportunities to experimental and clinical studies. Neuromuscular imaging should be crucial for emergence of studies concerning advanced neuroimaging technologies to predict movement disorders, postural imbalance with integrated application of imaging, and functional modalities for rehabilitation and pain management. Scientific results should initiate evidence-based preventive movement programs in sport medicine rehabilitation. Traditional medicine and mathematical analytical approaches and education challenges are discussed in this review. The physiological management of exactly assessed pathological condition, particularly in movement disorders, requires participative medical approach to gain harmonized and sustainable effect.

## Review

The problem of pain remains to be very common in the society, impacts on the disease intercourse, and patients' life quality. The concept for predictive, preventive, and personalized medicine cannot be entirely realized without consideration of adequate physiological integrative management of pain in medicine of the future. Existing treatment pain protocols leave a minor physiological relevance and personalized orientation (e.g., medication treatment). Pharmacological schemes are mostly directed to pain relief, do not suggest solution of the basic problem of pain treatment, and pose other challenges for the patient's health. Predictive schemes still lack in healthcare systems; the opportunities of routine clinical utilization of advanced technological approaches and analytics for diagnosis and prediction disorders, related to peripheral nervous system and neuro-muscular junctions, are still not applied commonly. Particular studies and practical approaches in these directions are mostly disintegrated. Despite the large number of preventive programs in rehabilitation and sport medicine, they require higher level of scientific basis for personalized adaptation. The novel predictive, preventive, and personalized medicine (PPPM) activity will increase a part of physically active contributing members of the society in elderly age in good physical and mental health [1,2], providing a complex performance of the proposed programs to actualize the ‘three-dimensional’ PPP vision in medical approaches declared by the European Association for Predictive, Preventive and Personalized Medicine (EPMA). Traditional medicine prevention strategies may provide some answers to the difficulties faced by healthcare systems considering the human being as a physical, psychological, and spiritual entity for individualized treatment algorithms and medical approaches tailored to the patient in the concept of person-centered medicine within EPMA [3-5].

### Myofascial pain—personalized assessment

In the structure of the pain morbidity, the myofascial pain is most notable. Most pain syndromes in clinical practice have a myofascial nature caused by the trigger point (TrP) and myofascial trigger point (MTrP) formation [6]. MTrPs are small hypersensitive areas in the skeletal muscle, fascia, tendons, and ligaments. They are painful when pressed and can irradiate a pain to other parts of the body. This can lead to abnormal sensitivity and vegetative phenomena, such as dizziness, numbness, and dysesthesia [7]. This trigger point can be either ‘active’ (trigger points that cause pain radiation during palpation) or ‘latent’ where palpation causes only a local hypersensitivity. Frequency of active MTrP reaches its maximum in the middle age. Elderly people reveal many latent trigger points. Women are more likely than men to visit a doctor because of myofascial pain. Travell and Simons [6] argue that normal muscle can contain MTrP, but these are not painful on palpation, do not cause the convulsive reactions, and do not reflect the pain due to its compression. According to Bates [8], myofascial MTrP is the main source of pain in skeletal muscles in children. In 1938, a British rheumatologist, Kellgren, published a description of specific patterns of reflected pain in the different groups of muscles and ligaments of the spine after injection of hypertonic saline solution. The term ‘trigger point’ was introduced by Steindler in 1940 [9]. Prior to this, in 1816, Balfour described painful inflamed nodules in the muscles. Since then, over the years, different terms have been used to describe trigger points: fibrosis myofasciitis, muscular rheumatism, rheumatic myositis, myogelosis, myalgia, myofascial pain, and fibromyalgia [10]. In 1983, Travell and Simons issued a classic two volume work titled *Myofascial Pain and Dysfunction*. After its second reissue in 1999, treatment of myofascial pain by acting on the trigger point was elevated to a higher, more modern level of expertise. In 1952, Janet Travell (1901–1997) published one of the first articles recognizing the specificity of reflected pain with models of more than 30 muscles [11]. As a pioneer in the treatment of musculoskeletal pain by MTrP determination, she introduced the term ‘myofascial pain syndrome’ to describe the pain generated from the trigger points in the muscles, tendons, skin, fascia, and ligaments. Several of her works were devoted to сraniomandibular pain [12,13].

### Personalization of interventional treatment of the trigger points

Dry needling of the trigger points, also called intramuscular stimulation, is an invasive procedure in which a needle (often an acupuncture needle) is introduced in the skin or muscles [14]. Trigger point dry needling (TrP-DN) is a relatively new technique often used in combination with other methods of physical therapy. Local injections have been used in different ways for decades, with publications on it dated from the early 1940s [15-17]. Today, dry needling of the trigger points has been approved in many physiotherapy protocols [18].

*Superficial dry needling (SDN)* is the introduction of a needle into the surface tissue to a depth of 5–10 mm directly above the palpable MTrP. In the early 1980s, Baldry [19], concerned about the risk of pneumothorax in patients with MTrP in the anterior scalenus muscle, advocated SDN. Instead of using TRP-DDN, he introduced the needle into the superficial tissue, just above the MTrP. After withdrawing the needle after a short time, the pain quickly and easily passed. Based on this experience, Baldry popularized the practice of SDN, inactivating MTrPs in different parts of the body with good empirical results, even in the treatment MTrPs of deeper muscles. He recommended the introduction the acupuncture needle into the tissue covering every MTrP to a depth of 5–10 mm for 30 s.

*Deep dry needling (DDN)* is the introduction of a needle directly into deep MTrP, causing the ‘local twitch response’ (LTR) effect of soreness in the course of pain radiation. This procedure requires manipulation of a needle and is considered to be a painful procedure (in blind performance) causing pain after the puncture. It can also be used in cases of nerve root compression by the deep muscle spasm.

DDN has been used for centuries, but a Czech doctor, Karel Lewit, was the first researcher in modern times to become a strong supporter of it. In his classic work, published in 1979 [20], he described the results of the treatment of myofascial pain in 241 patients where he introduces a needle into the zone of major sensitivity (trigger zones), ‘the pain point’ (as he termed it), which we now call MTrPs. Dr. Lewit acknowledged that this kind of deep dry needling leads to considerable pain relief but said that its effectiveness depends on the intensity of pain when the needle is inserted to a point. It also depends on the accuracy of verification of the trigger point for the puncture. Gunn [21] thoroughly investigated and popularized the analgesic effect of this type of treatment for myofascial pain syndromes. He called this technique ‘intramuscular stimulation’.

Deep dry needling is considered to be the best way to inactivate the trigger points. The action of the dry needle is the induction effect of local twitch response. The disadvantages are its low provability and possible complications due to the *inaccuracy of method*. Only personalized imaging-, model-, and intellectually guided approach should solve this *inaccuracy*.

### Visualization of the trigger points—new technologies are required

The technologies of imaging become more advanced continuing to move to higher frequencies in ultrasound (US) imaging and higher magnetic fields in MRI allowing much more accurate estimates of many of the predictive biomarkers. Additionally, hybrid systems, e.g., combined MR and positron emission tomography (PET) to more intimately relate morphology and function [22]. We previously reported trigger point dry needling, peripheral neuropathy, and local myopathy ultrasound diagnosis [23-26]. In the study from 2010 [23], new approach of the trigger point therapy was proposed, performing muscle punction under ultrasound guidance. According to this experience, the use of ultrasound imaging techniques significantly increases the detection accuracy and specificity of the verification of trigger points as the causes of myofascial pain and carries out the dynamic control of the effectiveness of the treatment. Untill now, palpation was the only method available for the clinical evaluation of myofascial pain.

Previously, there was no gold standard of pathological test for the identification of the trigger points. In 2007, using magnetic resonance elastography, trigger points were recorded as zones of reduced flexibility [27]. Minimally invasive ultrasound-guided soft tissue manipulations have become more important [28], and ultrasound navigation in minimally invasive musculoskeletal interventions gives significantly better effects than blind needle insertion based only on anatomical landmarks [29]. Kalichman emphasizes on the potential risk of significant adverse events, such as in the lungs and large blood vessels; we suggest using dry needling without visual navigation [30]. Botwin et al. issued a report concerning ultrasound-guided injections of the trigger points [31]. This technique was done to prevent complications (damage to blood vessels, nerves, esophagus, etc.) and improve the effectiveness of manipulation. In this study, the needle was inserted into the muscle under US control, but the trigger point was not identified. Wet needling rather than dry needling was used. According to the most recent studies, ultrasonography was considered to be helpful for detecting LTRs in deeper muscles that were missed on visual inspection [32]. Recently, studies emerge concerning the visual properties of myofascial trigger points. Thus, preliminary results from the study by Ballyns et al. indicate that patients with spontaneous neck pain and symptomatic myofascial trigger points have increased tissue heterogeneity at the trigger point site and the surrounding muscle tissue [33].

### Challenging methodology—trigger point dry needling under ultrasound guidance

**Figure 1:**
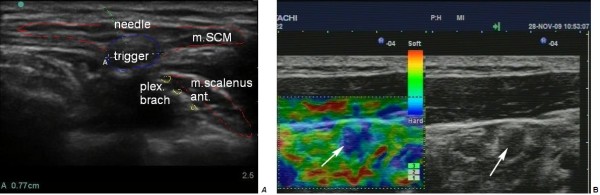
**Transverse scan and the trigger point.** (**A**) Transverse scan of the jugular region. Ultrasound visualization of the trigger points (trigger) and the needle inserted under ultrasound guidance. plex. brach, brachial plexus; m.SCM, sternocleidomastoid muscle; m. scalenus ant., anterior scalenus muscle. (**B**) The trigger point in anterior scalene muscle. Sonoelastography application depicts the blue area of rigid muscle tissue (arrows).

1. Clinical definition zone of possible trigger point—pain syndrome with typical referred pain pattern registration.

2. Trigger point palpation. Palpation of a hypersensitive bundle or nodule of the muscle fiber of harder than normal consistency. Localization of a trigger point is based on the sense of feel, assisted by patient expressions of pain, and by visual and palpable observations of local twitch response.

3. Using additionally other commonly used physical examination tests such as the assessment of intervertebral motion or paravertebral muscle strength testing (interrater reliability ranges from 41% to 97%) [3,4].

4. When the affected muscle is detected, ultrasonography examination is performed for myofascial trigger point visualization using gray-scale, Doppler, and sonoelastography (Figure 1).

5. After the visual identification of the trigger point, dry needling was performed—acupuncture needles were inserted into MTrP to elicit the LTR effect. The needle was held in the tissue until complete disappearance of the LTR which could be considered similar to the phenomenon of the ‘needle grasp,’ which has been attributed to the muscle fibers contracting around the needle, and was held tightly in place to increase the resistance to further move the inserted needle.

6. Ultrasound control after procedure.

7. Visual analog scale (VAS) scores (0–10) were recorded throughout the study period before, immediately after, and 24 h after the procedure.

### A comparison between the trigger point dry needling under ultrasound guidance and blind technique

**Figure 2:**
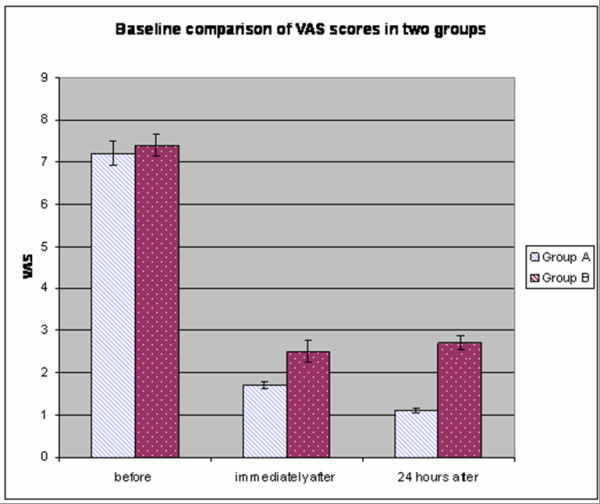
Diagram of VAS score changes in two groups.

The study [23] included 133 patients who were randomly assigned to either the dry needle trigger point therapy under ultrasound guidance, group A, or to the dry needle trigger point therapy using clinical (palpatory) established landmarks, group B. The pain relief effect (more than 50% of VAS decrease) was registered in all patients of the two groups. The pain measured using visual analog scale (0–10) showed an improvement from 7.2 to 1.1 at 24 h after the procedure in group A (pain level decreased to 84%) compared to the improvement from 7.4 to 2.7 at 24 h after the procedure (pain level decreased to 63.5%) in group B (*P* < 0.001) (see the diagram in Figure 2). Significant decreases were observed at average number of injected needles (2.6 ± 0.54 in group A compared to 4.45 ± 0.7 in group B). The level of LTR eliciting was 92.26 ± 3.8% in group A patients compared to 58.8 ± 7.5% in group B patients (*P* < 0.001). Average number of sessions was significantly lower in group A: 2.3 compared to 3.6 ± 1.7 in group B (*P* < 0.001). The dry needling of muscle trigger point under ultrasound control was performed. Study results are describe in Tables 1,2,3 and Figure 2.

**Table 1:** **Group A: results of trigger point visualization and inactivation by dry needling under US guidance** (***n*= 91**)

**Trigger point localization**	**Number of trigger points needled in one patient**	**Visualized trigger point**		**LTR elicited (%)**	**Average number of sessions**
		**By gray-scale US (%)**	**With sonoelastography (%)**		
Hand and wrist pain (*n* = 15)	1.5 ± 0.25	53	93.3	86.7	1.1 ± 0.25
Shoulder dysfunction (*n* = 45)	3.2 ± 0.25	71	77.8	88.9	2.8 ± 0.45
TMJ dysfunction (*n* = 8)	3 ± 1	38	25	100	3 ± 1.6
Rib dysfunction (*n* = 16)	1.5 ± 0.4	56	68.7	100	1.1 ± 0.25
Pelvic pain (*n* = 7)	4 ± 0.8	29	57	85.7	3.5 ± 1.1
*General*	*2.6 ± 0.54*	*49.4 ± 14.6*	*64.36 ± 22.4*	*92.26 ± 3.8*	*2.3 ± 0.73*

TMJ, temporomandibular joint.

**Table 2:** **Group B** (**controls**)**: results of trigger point dry needling without US guidance** (***n*= 42**)

**Research groups**	**Number of trigger points needled in one patient**	**LTR elicited (%)**	**Average number of sessions**
Hand and wrist pain (*n* = 5)	3 ± 0.25	73	2.4 ± 0.5
Shoulder dysfunction (*n* = 19)	5.2 ± 0.6	63	4.8 ± 0.4
TMJ dysfunction (*n* = 3)	4.7 ± 1	25	3 ± 1
Rib dysfunction (*n* = 12)	2.6 ± 0.3	100	2.2 ± 0.4
Pelvic pain (*n* = 3)	5.3 ± 0.8	47	4.3 ± 1
*General*	*4.45 ± 0.7*	*58.8 ± 7.5*	*3.6 ± 0.7*

**Table 3:** Baseline comparisons of the two groups

**Parameter**	**Group A trigger point visualization and inactivation by dry needling under US guidance** (***n*= 91**)		**Group B**, **controls** (***n*= 42**)	
	**Mean**	**SEM**	**Mean**	**SEM**
VAS before	7.2	0.4 (5.5%)	7.4	0.3 (4%)
VAS immediately after	1.7	0.1 (5.8%)	2.5	0.2 (12%)
VAS 24 h after	1.1	0.05 (4.5%)	2.7	0.2 (7%)
VAS changes	84%	5%	63.5%	7.7%
Number of needled trigger points	2.6	0.54	4.45	0.7
LTR elicited	92.26	3.8%	58.8	7.5%
Average number of sessions	2.3	0.73	3.6	0.7

SEM, standard error of the mean.

There were significant correlations registered in the two groups between the level of eliciting LTR during needling and the pain relief effect (VAS decreased more than the average percentage in group) (*r* = 0.717 and 0.67, respectively). Puncture of some groups of muscles in this study could not be possible without ultrasound visual navigation. In 34 patients of group A, regression of ultrasound symptoms of trigger point was registered. In eight patients (44%), which had undergone electromyograms (EMG) under US guidance, the spontaneous muscle activity was registered in all cases the eliciting of LTR was present.

### Minimally invasive treatment tools

Injections of local anesthetics have not achieved a better effect than the introduction of normal saline [34]. In this study, dry needling was just as effective as injections of local anesthetics such as procaine (Novocain), or lidocaine (Xylocaine). Dry needling and the introduction of 0.5% lidocaine were equally effective in relieving myofascial pain. Postinjection pain develops more often after using the dry needling technique.

When comparing the injection therapy with TrP-DN, many authors believe that the MTrP dry needling provides greater pain relief than the injection of lidocaine, but this also becomes a matter of greater post-injection pain. Typically, these authors refer to the study of Hong [35], who compared the effectiveness of injections of lidocaine with TrP-DN, but this author performed the injections of lidocaine and TrP-DN using conventional syringe needles rather than acupuncture needles. Cotchett et al. review the effectiveness of dry needling and/or injections of MTrPs associated with plantar heel pain [36]. Their first randomized controlled trial to evaluate the effectiveness of dry needling for plantar heel pain provided evidence for the effectiveness of trigger point dry needling for plantar heel pain [37]. Sarrafzadeh's study results indicate that pressure release, phonophoresis of hydrocortisone, and ultrasound were effective for treating upper trapezius latent myofascial trigger point [38]. There is limited evidence of botulinum toxin for the effectiveness of the treatment of MTrPs. According to the systematic review by Ho and Tan [39], among five clinical trials, one trial concluded that botulinum toxin A injection in the trigger points was effective and four concluded that it was not effective for reducing myofascial pain arising from the trigger points. Kamanli et al. [40] updated the study of Hong from 1994, comparing the results of lidocaine injections, injections of botulinum toxin, and TrP-DN. In this study, the researchers also used syringe needles, and they did not take into account the effect of LTRs. In clinical practice, using acupuncture needles to do TrP-DN is preferable. Gazi concluded that acupuncture, when compared with trigger point injection, combined with cyclobenzaprine chlorhydrate and sodium dipyrone provided similar pain relief and improvement in quality of life measures at 4 weeks [41].

The assumption, based on published studies, that TrP-DN would cause great pain after needle insertion compared with injections of lidocaine cannot be objective, since the latter would not have arisen if acupuncture (fine) needles were used. In studies conducted to determine the optimum diameter of the needle, it is believed that at least 21- to 23-gauge diameter needles are optimal to do dry needling [32]. Itoh et al. came to the conclusion that the DDN can be more effective in the treatment of low back pain in elderly patients than standard acupuncture or SDN [4]. Cummings and White concluded that “the nature of the injectable substance is irrelevant to the outcome of treatment, and injections (‘wet needling’) has no therapeutic advantages over dry needling” [11].

Recently, we performed a comparative study [24] in 44 patients between trigger point dry needling and injection of local anesthetic with the application of ultrasound guidance for the shoulder myofascial pain treatment after exclusion of rheumatic, neuropathic pain. Our results showed that *MTrP dry needling is significantly preferred over injection treatment if reliable ultrasound guidance is provided*, evokes higher pain relief, and lowers pain and spasticity recurrence at 24 h after the first procedure and better long-term outcome.

### Personalized management of local muscle spasticity in different patient groups

In addition, the most recent studies support the statement that US guidance notably improves injection accuracy in the musculoskeletal interventions and directly improves patient-reported clinical outcomes and cost-effectiveness [42]. However, according to Huang [43], treatment outcome depends not only on the dry needling protocol but also on disease characteristics and patient demographic profile.

**Figure 3:**
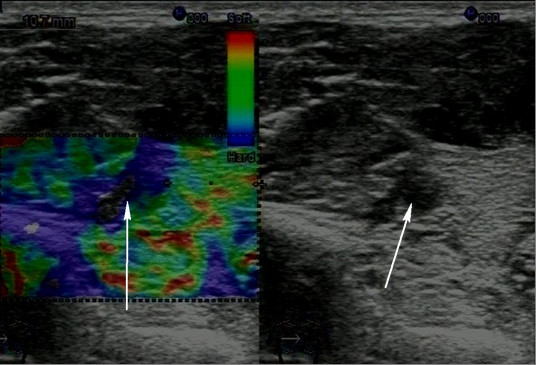
**Ultrasound visualization of the affected muscle in patient with hereditary myopathy.** The case shows contracted muscle tissue (hypoechoic) vs. background of increased tissue echogenicity (bright) (arrows).

The results of inactivation of the TrPs as areas of contracted muscle fibers are reproducible for the treatment of other types of local muscle spasticity. Thus, we performed the study [44], that included recruiting 36 patients, who suffered from local muscle spasticity that caused movement, postural disorders, and pain with different pathology, including hereditary myopathy, Parkinson's disease, rheumatic (dermatomyositis and sclerodermia), peripheral neuropathy, poststroke spasticity, and trigger points. Patients underwent US-guided needle-EMG, biopsy for diagnosis and dry needling for the treatment of local spasticity. In all patients, there were registered changes on ultrasonography. Local spasticity of the muscle was visualized as areas of violation of the normal fibrillar structure of affected muscles, mostly hypoechoic (Figure 3). Spontaneous fibrillation was clearly visualized as well as the specific changes of echogenicity and stiffness of the affected areas. Functional tests were performed to identify areas with increased susceptibility to spontaneous contractile activity. Sonoelastography identified areas of decreased elasticity in the muscle. Integrated application of neuromuscular US recognized specific patterns, and US-guided neurophysiologic study and biopsy allow to recieve additional information. Dry needling was effective for inactivation of the trigger points (94%) and had short effect in 75% patients with Parkinson's disease [44]. 

Yamaguchi [45,46] considers TrP therapy to be very useful for the treatment of myofascial pain in cancer patients. The positive outcome in comorbid symptoms and overall quality of life was reported in a patient with rheumatoid arthritis and collagenous colitis, after application of sustained release MFR techniques in addition to standard medical treatment [47].

### Personalized management of temporomandibular joint dysfunction and craniomandibular pain syndromes

While ultrasonography application improves the effectiveness and safety of deep dry needling as an optimal method of the trigger point inactivation for myofascial pain treatment, it is effective for management of temporomandibular joint dysfunction and craniomandibular pain syndromes. The temporomandibular joint (TMJ) is part of a kinematic chain including the teeth, the opposite TMJ, the muscles of mastication, and the upper cervical complex, as well as the posterior cervical musculature. In this context, temporomandibular joint dysfunction (TMJD) refers to a group of non-specific related disorders of the muscles of mastication and the TMJ but excludes non-musculoskeletal disorders in the orofacial region such as neoplastic, vascular, or infectious diseases that produce very similar symptoms. In this regard, it is estimated that 85%–90% of the population will develop some symptoms of TMJD in this form at some point during their life, and research has shown that the prevalence of females to males seeking treatment will be 3:1 [48]. Physically, the stomatognathic system is composed of anatomic elements, including the teeth, TMJs, and the muscles [49]. Therefore, abnormal function or loading on one element can cause changes in other elements in the system. For example, asymmetries of the maxillofacial skeleton and occlusion can produce changes in mandibular posture [50] or asymmetric mandibular function [51].

Functional relationship with dysfunction of the spine should be considered in patients with craniomandibular pain syndromes; knowledge of myofascial pain has been constant in dental community [52-54]. The application of dry needling into active TrPs in the masseter muscle induced significant increases in pressure pain threshold levels and maximal jaw opening when compared to the sham dry needling in patients with myofascial temporomandibular disorders [55].The findings of the study by Gonzalez-Perez et al. suggest that deep dry needling in the trigger point in the external pterygoid muscle can be effective in the management of patients with myofascial pain located in that muscle [56]. Rocabado developed a comprehensive approach to treat TMJ dysfunction, in view of complex relationship between the cervical spine, mandible, and function of temporomandibular joint. He demonstrated that the centralization of the position can be achieved only if there is a balance between the structural and motor patterns of the subcranial area, the middle and lower cervical spine, the hyoid bone, and the mandible [57,58]. The majority of authors' report have specifically reflected the pain patterns in masticatory muscles [59,60].

However, many clinical conditions are included in the differential diagnosis of myofascial pain associated to the trigger points. In chronic cases, psychosocial intervention is required due to the high incidence of mood disorders and/or anxiety observed in these patients, who in turn present a poorer prognosis [52].

### Three-dimensional model-guided needling

**Figure 4:**
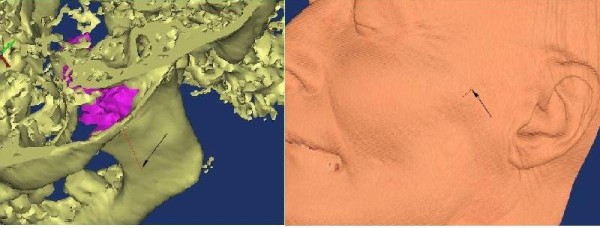
**Models of needle direction for medial pterygopalatine muscle puncture.** Left, on the muscle-bone model; right, projection on the skin. Fine needle is indicated by arrows.

**Figure 5:**
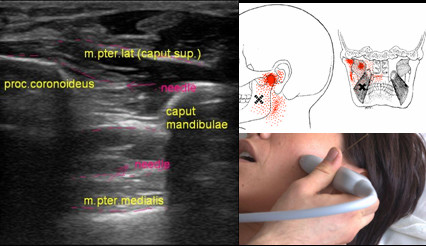
**Scheme of the irradiation pattern, ultrasonogram, and puncture.** Irradiation pattern of the pain (right top), ultrasonogram of needling trigger points in the medial pterygoid muscle (left). m.pter.lat (caput sup.), lateral pterygoid muscle (superior head); proc.coronoideus, coronoid process of the mandible; caput mandibulae, head of the mandible; m.pter.medialis, medial pterygoid muscle. Puncture of the lateral pterygoid muscle under ultrasound guidance (right bottom).

Terajima et al. reported about the four-dimensional (4D) analysis of stomatognathic function that combines the three-dimensional (3D) computed tomography of the cranium and mandible, dental surface imaging with a non-contact 3D laser scanner, and mandibular movement data recorded with a 6 degrees of freedom jaw-movement analyzer [61]. We apply the three-dimensional modeling based on ultrasound data segmentation and conjoining the models [62] with those created from different source data of visual information (CT, MRI, and photogrammetry) in a single three-dimensional environment for planning intervention under the ultrasound guidance in real time. The three-dimensional modeling becomes a base of initiation for the model-guided interventions on difficult locations of the trigger points (Figures 4 and 5).

Using trigger point dry needling under US guidance, the pain was eliminated; the efficient correction of the occlusion, which was previously impossible due to spastic phenomena in the muscles, was performed. The correctly performed procedure can be relevant for the prevention of posture disorders, pain in cranio-mandibular region, and dysfunction of TMJ.

### Personalized pelvic pain treatment

Myofascial pelvic pain is a widespread problem, occurs more frequently in women, and detected in 10%–15% of all gynecological patients. Recently, we proposed a new approach of trigger point therapy performing precise muscle dry needling under US guidance. The method of dry needling trigger points under ultrasound guidance can be considered as an effective practice for the treatment of the idiopathic pelvic pain, evoked by myofascial disorders [22]. Our study included seven patients (females); average age was 68 ± 7 years old. All patients suffered from extensive pelvic pain with different locations, all with vulvodynia. Main active trigger points were diagnosed in deep pelvic muscles that caused compression of the n.pudendus in Alcock's canal. All patients showed decrease in pain as measured by a VAS of 64%; the difference was significant in this group (*p* < 0.01), and pain relief outcome was after 1-month observation.

### Assessment of peripheral neuropathy—promising predictive modalities for diabetes and neurodegenereative diseases

**Figure 6:**
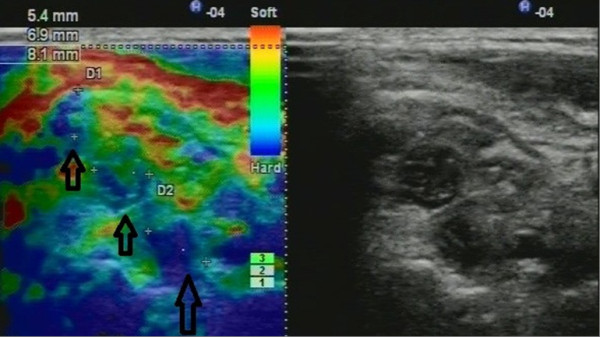
**Ultrasound of brachial plexus neuropathy.** Arrows indicate enlarged (swollen) brachial plexus trunks up to 8 mm (contralateral trunks were about 3 mm). Sonoelastography (left) indicates rigid tissues of trunks (colored blue). Gray-scale ultrasonogram (right).

The visualization previously has not been used widely to diagnose neuropathy, but diagnostic ultrasound is used for peripheral nerve visualization, particularly in regional anesthesia. We performed the study aiming to formulate a diagnostic marker of neuropathy [63]. Thus, normal US criteria of lower and upper extremity nerves were established. The ultrasound nerve structure changes in all patients with neuropathy were found. These findings were made possible by the establishment of empirical ultrasonic neuropathy symptoms obtained in assessing the structure of the nerve (Figure 6). Also, the few US patterns of neuropathy may be distinguished. US is a relevant alternative diagnostic method for neurophysiological studies. The combination of clinical, sonography, and US-guided electromyography can reliably estimate the peripheral nerve diseases. These novel clues make opportunities for neuropathic pain management. 

### Trigger point inactivation mechanism—crucial issue for physiological pain treatment

As we observed, in patients who underwent treatment using ultrasound guidance, the pain relief effect significantly increased, and the average number of needled trigger points and average number of treatment sessions significantly decreased. There were significant correlations registered between eliciting LTR during needling and the pain relief effect.

The question of evidence and effectiveness of trigger point therapy depends on accurate detection of active trigger points and direct introduction of a needle into the trigger. Thus, in this study, it is acceptable to declare that US imaging is an effective and necessary modality in myofascial pain treatment.

In order to further discuss the method of dry needling using ultrasound guidance development, it is necessary to clarify a few statements. If the effect is the same, what is the reason for injecting any substance? The principal findings of the latest reports indicate that pain treatment outcomes do not depend on the nature of the injected substance into the myofascial trigger point and that wet needling is not therapeutically superior to dry needling. These conclusions are supported by several series of studies [31,40,64]. Real-time imaging studies are promising methods for the solution of the task of muscle disorder mechanism.

The trigger point is inactivated by either injection or strong MTrP palpation/finger compression, which leads to a brief rupture of motor action potentials—available only on constricted fibers. There has been a study made with evidence indicating that dry needle-evoked inactivation of a primary (key) MTrP inhibits the activity in satellite MTrPs situated in its zone of pain referral. This supports the concept that activity in a primary MTrP leads to the development of activity in satellite MTrPs and the suggested spinal cord mechanism responsible for this phenomenon [65]. The study of Tsai et al. demonstrates the remote effectiveness of dry needling of a distal myofascial trigger point that can provide a remote effect to reduce the activity of a main and proximally located myofascial trigger point [66]. *For this reason, it is possible to decrease the amount of injecting needles.*

However, the issue of MTrP relevant inactivation is a very challenging task [3]. The discussion between the efficiency of wet and dry needlings remains unfinished. Our results show that dry needling of MTrP can be preferred over injection treatment if reliable ultrasound guidance is provided [41].

### Hypothesis: LTR could be possibly be a treatment mechanism

It is known that MTrP dry needling is most effective after LTR is elicited [35]. Deep dry needling involves inserting a needle into the center of a TrP in order to elicit one or multiple LTRs [67]. The change in the length of the fibers caused by the LTR is thought to stimulate mechanoreceptors with large diameter, fast-conducting fibers. Hong has demonstrated that either injection of a local anesthetic agent into MTrP or dry needling carried out on it is similarly effective in alleviating MTrP pain [68].

A number of authors [69,70] have suggested that the greater the amplitude of the LTR, the greater the pain relief afforded by the needling treatment. These opinions were based upon clinical observation but need to be confirmed by experimental evidence. It could be suggested that the degree of irritability is proportionate to the number of LTR loci (sensitized nociceptors) in the MTrP region. Notwithstanding this deficiency, this postulate has provided a theory for the therapeutic relief associated with the LTR and the belief that LTRs may be the key to pain relief, rather than just a diagnostic sign for the localization of TrPs. For this reason, Hong [68] and Chu [69] strongly support obtaining multiple LTRs in treating TrPs, believing that, doing so (by rapidly re-inserting the needle into the TrP region), this increases the effectiveness of DDN.

In humans, stimulation of the TrP locus can elicit pain and LTR. However, when the MTrP is hyperirritable, even low-pressure stimulation can elicit referred pain and LTR [71-73]. In other words, in terms of post-treatment pain reduction, those who experienced most discomfort during needle stimulation (movement of the needle, possibly eliciting LTRs, though not reported) experienced greater treatment responses. This suggests that the intensity of needle stimulation, that is, the amount of needle movement, should be taken into consideration when applying dry needling techniques in order to increase the muscle blood flow in chronic pain conditions.

Clinically, there is a slight increase of muscle spasm, after which it decreases [74]. A physical characteristic of collagen fibers is their intrinsic piezoelectricity, a property that allows tissues to transform mechanical stress into electrical activity necessary for tissue remodeling [75]. Travell and Simons indicate that the therapeutic effect of TrP-DDN was mechanical disruption of the MTrP contraction knots [3]. Langevin et al. [76-80] described the phenomenon of the needle grasp, which has been attributed to muscle fibers contracting around the needle and holding the needle tightly in place. During the needle grasp, a clinician experiences an increased pulling of the needle and an increased resistance to further movement of the inserted needle. To provide ultra-localized stretch to the contracted structures, it may be caused by rotating the needle. An accurately placed needle may also provide a localized stretch to the contracted cytoskeletal structures, which would allow the involved sarcomeres to resume their resting length by reducing the degree of overlap between actin and myosin filaments [81].

Bron [82] reports the high prevalence of muscles with latent MTrPs and the association between MTrPs and the severity of pain and functioning in patients with chronic non-traumatic unilateral shoulder pain. A latent MTP is associated with an accelerated development of muscle fatigue and simultaneously overloading active motor units close to an MTP. Elimination of latent MTPs and inactivation of active MTPs may effectively reduce accelerated muscle fatigue and prevent overload spreading within a muscle [83].

Postural disorders often contribute to the perpetuation of TrPs. The phenomenon of reciprocal inhibition and protective spasm for the entire synergistic muscle groups or singular muscles and soft tissue structures is very likely to be a main mechanism of the TrP appearance. This phenomenon is used in manual medicine techniques that utilize reflexive antagonism [84]. ‘Intrafusal muscle fibers’ are innervated by gamma motor neurons and are a proprioceptor that detects the amount and rate of change of muscle length. Contraction of these fibers might be a cause of the trigger points [85]. The Golgi tendon reflex protects the skeletal muscle by causing its relaxation. It is possibly included into the mechanism for trigger point inactivation [86]. *Thus, we consider that any trigger point, despite the cause of its appearance, should be inactivated to avoid the progression of postural imbalance and neurosomatic consequences. Even after the elimination of initial background, the trigger will persist as a latent or active one.*

### Depth of needling

However, in acupuncture, it has also been suggested that the insertion of needles may have a therapeutic effect more or less irrespective of the needling site, depth, or stimulation [87]. The heterogeneity of acupuncture treatments has been used to support the argument that acupuncture works predominantly or entirely as a complex placebo [88]. While applying US-guided dry needling, the issue of depth of needling is defined by the depth, where the TrPs is recognized, since it is possible to clearly visualize TrPs in depth about 5 cm and more. It is acceptable for the treatment of most possibly involved muscles. The question is about the relatively rare need to use needles for more deep needling, looking for approach that is more preferable (due to the shorter distance). In cases for ‘extra deep’ needling, the application of additional imaging modalities as a vector of 3D modeling and the use of special intracavital approaches are helpful.

### Morphology and substrate of trigger point for visualization

As an analogy to the researched symptoms of trigger point ultrasound reflection, it is important to consider the changes in the muscles, which result in myopathy. Few studies have been previously performed [89,90], but according to these studies, ultrasound in patients with muscular dystrophy exhibits uniform increase in echogenicity of the tissue, while the secondary muscular atrophy shows heterogeneous ultrasound ‘moth-eaten’ pictures of the dark (hypoechoic) and light (increased echogenicity) plots. We described the method in [91], considering the muscle tissue ultrasonographic phenomena for diagnostics of myopathy as follows: the affected muscle has increased echogenicity, the contracted muscle tissue is less echogenic, the sonoelastography shows a lesser elasticity of the contracted muscle than the adjacent areas of the relaxed muscle, and the affected areas of the muscle have a reduced ability to stretch compared to the unaffected areas while performing stretching functional tests. After functional tests, the affected areas reveal spontaneous fibrillar activity. Muscle biopsy under ultrasound guidance shows degenerative changes; precise electromyographic study under ultrasound detects specific changes.

In neuromuscular disorders and in cases of long-term trigger point persistence, the affected muscle is replaced by fat and fibrous tissue, resulting in the increased density of limited areas with different acoustic impedances and increased reflection of the ultrasound beam. Biochemical differences were found by Shah and Gilliams [92] between active and latent MTrPs as well as in comparison with healthy muscle tissue.

### Neurophysiology pain study: electromyography

**Figure 7:**
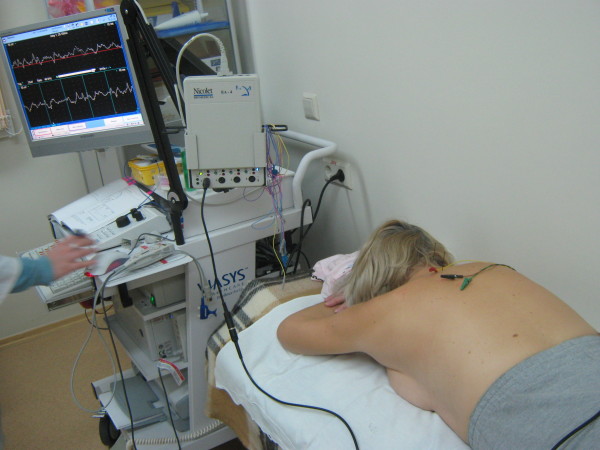
**Spontaneus muscle activity in the trigger point**, **needled with US guidance**, **while LTR effect is evoked.**

Hubbard and Berkoff studies indicate that monopolar needle EMG showed sustained spontaneous EMG activity in all TrPs and was absent in non-TrPs. The authors hypothesize that TrPs are caused by sympathetically activated intrafusal contractions [93]. It was suggested by Gemmell [94] that there is a trend for muscles containing active trigger points to have less electrical activity than muscles containing latent trigger points. We obtained the preliminary results that direct needling EMG under US guidance that showed different spontaneous activity of the trigger points (Figure 7). A needle EMG study from nearby the trigger tissue remained electrically silent. We suppose this depends on the activity of the trigger point and the accuracy of needling to the trigger point. A combination of these approaches gives new opportunities to experimental and clinical studies. 

### Neurophysiology pain study: functional brain imaging in pain

By Travell and Simons' view, the central nervous system is an ‘integrator’ of TrPs and an ‘organizer’ of somatic dysfunction; the sensitized dorsal horn becomes a ‘neurologic lens,’ consolidating other nociceptive signals converging on the same segment of the spinal cord, including other somatic dysfunctions and visceral dysfunctions [95]. Over the last few years, remarkable efforts have been made using functional imaging studies to decipher underlying neuronal mechanisms of pain, resulting to deeper understanding of the basic human neurophysiology of pain and a potential neural framework for better pain management [96-103]. These studies were noticeably initiated by Wilder Penfield who described the results of stimulating the cerebral cortex in 163 awake adults undergoing intracranial surgery [104]. The techniques of positron emission tomography (PET), functional magnetic resonance imaging (fMRI), and single photon emission computed tomography (SPECT) are applied to the study of human pain processing and the assessment of physiological interventions or psychological manipulations [96]. The basic regional activation pattern after acute nociceptive stimulation is now fairly well clarified [96-99]. Maihöfner et al. [97] used fMRI and explored brain activation patterns during acute impact pain and mechanical hyperalgesia to study the central mechanisms of clinical neuropathic pain. One of the most distressing symptoms of many neuropathic pain syndromes is the enhanced pain sensation to tactile or thermal stimulation (hyperalgesia); there is a difference in the brain areas modulated by analgesia and antihyperalgesia.

In spite of systematic investigations, the existence of a specific cortex that could encode for the intensities of somatosensory stimuli, including within nociceptive ranges, is still a matter of debate. Peyron et al. suggest [98] the operculo-insular area as the only area in the brain to respond to the characteristics of a primary thermosensory and nociceptive cortex. Bentley et al. demonstrate the cingulate region location of the source in pain processing [99]. Pain intensity-related changes have been identified in the bilateral brain system including the parietal, insular, cingulate, and frontal cortical areas, as well as the thalamus, amygdala, and midbrain [100]. Specific patterns of activity may characterize hyperalgesic states and some chronic pain conditions.

Notably, a growing number of functional neuroimaging studies have demonstrated striking similarities (as well as differences) in the neural circuits involved in the processing of both the first-hand experience of pain and the second-hand experience of observing other individuals in pain [101,102]. Anticipation of pain may in itself induce changes in brain nociceptive networks. Moreover, pain-related cortical activity can be modulated by hypnotic suggestions, focusing or diverting attention, and placebo. These findings begin to disclose the spatio-temporal dynamics of brain networks underlying pain perception and modulation.

Boecker et al. [103] published findings using a positron emission tomography which support the ‘opioid theory’ and suggest region-specific effects in the frontolimbic brain areas that are involved in the processing of affective states and mood in runners, level of euphoria, correlated with opioid binding in prefrontal/orbitofrontal cortices, the anterior cingulate cortex, bilateral insula, parainsular cortex, and temporoparietal regions.

### fMRI and acupuncture

Classical acupoints are commonly used for modulatory and pain-reducing actions. The issues of fMRI investigation during acupuncture are important to demonstrate the correlation between the activation of specific areas of the brain cortices and the corresponding acupoint stimulation predicted by traditional acupuncture literature. Thus, Chinese review by Zhong et al. [105] shows that the development of researches on acupuncture therapy with fMRI focuses on the immediate efficacy and the specificity of efficacies of acupoints. New test design paradigms are agreeable with clinical practice; the stability of the tested results with the actuality of sustained effect of acupuncture is necessary in the future. *However, studies related to functional brain imaging for myofascial pain, emerging of active trigger point latent while performing guided manipulation, and eliciting LTR were not still initiated. Further functional brain imaging pain studies of cognitive, emotion, and visceral interrelations, combined with trigger point detection and treatment, might be crucial in determining the prior causes of trigger point appearance.*

### The clarification of terms of needling techniques

#### Trigger points and fibromyalgia

Currently, the term ‘fibromyalgia’ is widely used in evidence-based medicine as well as often in cases of a myofascial pain syndrome [106]. It is necessary to distinguish between the fibromyalgia syndrome (FMS) and the myofascial pain syndrome (MPS), which belong to the group of chronic non-inflammatory pain syndromes affecting the muscles and tendons. The presence of ‘tender points’ and ‘trigger points’ is an important sign in the diagnosis of both diseases. According to the criteria of the American College of Rheumatology, FMS is characterized by the presence of tender points and trigger points that are usually defined in the MPS. The term tender points is also used as diagnostic marker for the fibromylagia diagnosis. Tender points are the extremely sensitive points of the body, painful under compression weight of 4 kg (enough to make a nail pale in color). According to the American College of Rheumatology [107], the FMS is based on two main criteria: (1) the presence of a symmetric generalized pain (extending to the right and left, upper and lower half of the trunk or axial) which lasts longer than 3 months and (2) prevalence by palpation of at least 11 out of 18 (9 pairs) specific sensitive points.

Giamberardino reported that in patients with fibromyalgia showing local extinction of TrPs producing significant relief of FMS pain suggests that assessment and treatment of concurrent TrPs in FMS should be systematically performed before any specific fibromyalgia therapy is undertaken [108]. Alonso-Blanco et al. demonstrate the high prevalence of active TrPs in multiple muscles of fibromyalgia patients and the correlation between TrP activity and FMS pain and hypersensitivity. Widespread mechanical pain hypersensitivity was related to a greater number of active MTrPs [109].

### Traditional medicine approach

Melzack et al. [110,111] found that 71% of the trigger and acupuncture point localizations is the same. Most recently, Dorsher [112] compared the anatomic and clinical relationship between the 255 MTrPs described by Travell and Simons and 386 acupuncture points described in the Shanghai College of Traditional Medicine and other acupuncture reports. He believes that 92% of the 255 trigger points correspond to acupuncture points and that in 79.5% of them, they are identical due to the clinical indications in pain syndromes [113]. Dorsher concluded that there is considerable overlap between MTrPs and acupuncture points and claimed that ‘a high degree of consistency between the treatment of trigger points and acupuncture should contribute to strengthening the integration of acupuncture into contemporary clinical management of pain.’ Although these studies prove the feasibility of treatment of TrP-DN as a form of acupuncture, both studies suggest that there are different anatomical localization MTrPs, while acupuncture points have anatomical specificity. Dommerholt systemized the differences between both approaches [18,81].

Comparing the eliciting LTR in TrPs while dry needling and the needle grasp in acupuncture procedures, analogies between both are obvious since the early classic Chinese medical texts stating that the early detection of correct needle position is focused on the needle grasp by the acupuncturist's perception. Thus, Park et al. [114] published preliminary results, indicating a strong connection between acupuncture sensation and both the tissue depth and needle rotation.

The interventional imaging in orthopedic rehabilitation using traditional medicine approach is practiced while restoring the flow of energy (‘qi’) along the meridians according to traditional Chinese medicine and/or traditional Tibetan, Korean, or Japanese medicine, which is considered as aim of the treatment. When performing TrPDN, the practitioner has no direct intention to influence the meridians' energy. TrPDN is based on modern Western scientific principles and knowledge of anatomy and physiology.

Knowing that the term ‘dry needling’ has become a special issue in the acupuncture society, the dilemma of these terms is still actively discussed. We acknowledge the statement that acupuncture describes any stimulation technique (as titled), so dry needling might be a part of it, when following Simonds' and Travel's approach. The practice that is proposed here is the direct visualization of a trigger point and its precise needling. Trigger is the object of this study; thus, it is necessary to use such term as trigger point here. It would be also appropriate to keep both terms, ‘acupuncture’ and dry needling, because of further studies concerning US-guided meridian points in acupuncture. There also exists wet needling (injections) of the trigger points (this is not acupuncture). We must determine all possible methods.

The practice of acupuncture using imaging control and following clinical trials are strongly required, believing that imaging (mainly direct US in real time), conjoined with other (neurophysiological) studies, will give us an explanation of energy flow. This is necessary to be able to make some bridges between the different styles. This is a fascinating approach and invites further research. In practice of TrP-DN, most likely the energy flow was restored in meridians by inactivating TrP, but it *is not the intention of the intervention*.

Much closer to traditional Chinese acupuncture are positioned techniques, connected with tender (*ah shi*) point needling [115], that have a permanent location as well as acupuncture points the stimulation of the Chapman reflex points (or Chapman's points). These points are described as small, discrete tissue texture changes located just deep to the skin [116] that activate visceral reflexes to the internal organs. In order to distinguish these approaches, the term TrP-DN-US can be used.

We consider the traditional Chinese acupuncture and the employment of meridian points under ultrasound guidance (Acu-US) in practice. This technique is useful while performing deep acupuncture of points positioned near the vessels. Our experience in interventional ultrasonography and regional anesthesia under ultrasound guidance convinces us that the variable anatomy of the nerves and vessels makes necessary a visual (mostly ultrasound) identification of the inserted needle. There are pilot studies concerning this approach [117].

### TrP-DN-US vs. TrP-DN

Trigger point dry needling under ultrasound guidance (TrP-DN-US) is considered to be a separate methodic. It may belong to interventional sonography as well as orthopedic manipulation, etc. It is not the same technique as only TrP-DN because of its evidence and its higher effectiveness of application. Techniques of TrP-DN-US can be clearly protocoled, and educational standards can be determined. The object of treatment in TrP-DN-US is a very small area of the trigger (few millimeters only), and from our experience, *the consideration that it may be needled with effective inactivation without US navigation is very confusing*. It is especially difficult when performing deep dry needling.

Whether TrP-DN could be considered a form of acupuncture depends on how acupuncture is defined [81]. According to the position statement on TDN by the American Association of Acupuncture and Oriental Medicine [118], *Acupuncture as a technique is the stimulation of specific anatomical locations on the body, alone or in combination, to treat disease, pain, and dysfunction. Acupuncture as a technique includes the invasive or non-invasive stimulation of said locations by means of needles or other thermal, electrical, light, mechanical, or manual therapeutic method.* TrP-DN-US may be acupunctured due to the stimulation mechanism, but at the same time, acupuncture is not TrP-DN and TrP-DN-US. Due to this definition, stimulation techniques may include acupuncture. At the same time, there is a reason to distinguish *stimulation techniques of interventional ultrasonography* as TrP-DN-US, needle electromyography under ultrasound guidance [119,120], ultrasound-guided implantation of peripheral nerve stimulation [121], etc. In one pain management protocol, the techniques of regional anesthesia for continuous pain relief can also be used [122]. Regional anesthesia under ultrasound guidance and other injection techniques are commonly practiced and do not belong to acupuncture.

### Mathematical modeling approach for PPP pain medicine

According to expert recommendations from World EPMA Congress 2011 in Bonn, research and development on functional interactive models for an individual patient's health, allowing to predict potential health problems under diverse model situations [123], probabilistic graphical and related models enabling a mathematically structured integration of patient information from various independent sources in real time [124], were recommended to implement in the concept of model-guided medicine.

As most processes found in medicine are nonlinear, chaotic, and have a high level of complexity, creating a reliable mathematical model and use of information technology at all stages of the treatment process from the expression of the pathological processes to the implementation of therapeutic interventions associated with patient and physician perception of these phenomena, making decisions in the absence of input parameters for creation self-controlled systems based on forecasts of future medical errors are important tasks [125]. Previously, we reported [126] the approach solving combinatorial (correctable) problem of selection options of negative prognostic indicators for interventional radiology/ultrasonography mistakes to ensure a high level of patient safety as well as study-level skills and minimal training required for training programs for interventional medicine (in particular in pain management) by applying the stochastic method of branches and boundaries.

### Education challenges

Conducting minimally invasive interventions under radiology/ultrasound control requires continuous improvement of multidisciplinary approach to the analysis of errors and develops a differentiated approach to each clinical situation to achieve the efficiency of about 100%. We suggest designing the complex phantom-virtual training systems as a major component of the educational process in pain medicine. It should be a link between theoretical training and acceptability of specialist to work with the patient and also an effective tool for pre-manipulative planning in specific clinical situations and for new interventional technique implementation to provide high level of patient safety.

### Future outlook and recommendations

New paradigm requires future trials to use rigorous blind and randomized controlled methodology with an objective measurement, agreeable with clinical practice for long term observation.

Further studies of the following topics are required:

• meridian–trigger correlation study, approved by imaging techniques;

• trigger point image-guided biopsy and histology study;

• study on the improvement of imaging techniques;

• study of muscle contraction physiology;

• study to decrease the number of needling sessions and the number of trigger needling, and the necessity of the latent trigger point inactivation;

• study of the sequences of basic and latent trigger point needling and study of the mechanisms of postural compensation;

• creation of mathematical models of postural mechanism, neuromuscular networks, muscle–emotion–visceral relationships;

• study on virtual modeling of behavior stereotypes;

• study on the consequences of mistakes of needling in the wrong point using reliable mathematical models;

• study for standardization techniques and relevant educational program creation with virtual phantom trainer development;

• randomized controlled studies for the assessment of TrP locations, appearance, and interactions based on imaging-guided techniques;

•to study the possibility of advanced neuroimaging techniques to predict movement disorders, postural imbalance for integrated application of imaging, and functional modalities for rehabilitation and pain management;

• to study and promote the issues of appropriate education for physicians, practicing complementary and alternative medicine (CAM), in view of integrated CAM to the guidelines in Europe health care policy, granting strategies for this in general and particularly in pain and rehabilitation;

• adhere CAM-related trials to the Standards for Reporting Interventions in Controlled Trials of Acupuncture (STRICTA).

### Consolidation of the PPPM concept

#### Personalized medical approach

Following the concept that the particular clinical case of pain with inherence of all multiplicity of causes for its initiation is personally exceptional and requires treatment, adapted to the personal patient's condition. It is not managed by generalized standard protocols including pharmaceutical and minimally invasive approaches. Ultrasound may be considered as the most acceptable technique for image guidance and monitoring pain treatment. The integrated application of three-dimensional modeling based on the data from different sources of visual information with motion analysis improves the quality of diagnostics and pain syndrome treatment and leads to the performance of the model-guided interventions.

#### Predictive medical approach

Integration of the pain study and management techniques, perfection of diagnostic methods for the identification of pain source, including functional and imaging modalities, mostly clinically guided and patient related as, e.g., ultrasound with relevant medical record, further developing the experimental protocols and clinical studies by means of genetic, anatomical, histological, and physiological criteria, are required to increase the level of evidence and triggering standard educational programs.

Combination of the approaches of ultrasound and EMG gives new opportunities to experimental and clinical studies. The complexity of its emergence and regression of pain requires personalized intellectual image-guided techniques. Correct application of the stimulation techniques and development of experimental protocols and clinical studies with anatomic uses, histologic, and physiologic criteria are all required to increase the level of its evidence base on standardization of diagnostic methods of identification, including EMG and imaging as ultrasound, MRI/fMRI, PET, and SPECT studies.

#### Preventive medical approach

Neuromuscular imaging could be crucial for the emergence of studies concerning advanced neuroimaging technologies for prediction of movement disorders and postural imbalance. Integrated application of imaging and functional modalities is an innovative clinical application for pain development informative predictive biomarkers. Adequate inactivation of the trigger point will avoid the appearance of visceral, neurological consequences inherent to myofascial pain and postural imbalance. Scientific results should initiate scientifically based preventive movement programs in sport medicine rehabilitation.

#### Participative medical approach

The physiological management of exactly assessed pathological condition especially in movement disorders requires extended participation of patient to gain harmonized and sustainable effect.

## Conclusions

Concluding the declared points, we can formulate the following proposals (expert recommendations):

1. for *Europe*: initiation of topic-related multidisciplinary trials. To create an integrated concept of application of the imaging and functional modalities for rehabilitation and pain management with appropriate educational programs. We suggest designing the complex phantom-virtual training systems as a major component of the educational process in pain medicine. It should be a link between theoretical training and readiness of specialists to work with the patient and also an effective tool for pre-manipulative planning in specific clinical situations and for new interventional technique implementation to provide high level of patient safety.

2. for *Ukraine*: to organize teaching courses for peripheral nerve system including nerve conduction study, electromyography combined with ultrasound imaging application, and guided interventions, and participate in topic-related high-quality international multicenter trials.

## Consent

Written informed consent was obtained from all the patients for publication of this report and any accompanying images.

## Competing interests

The author declares that he has no competing interests.

## Author’s information

RVB is a Ph.D. holder and a medical doctor in the Centre of Ultrasound Diagnostics and Interventional Sonography, Clinical Hospital ‘Pheophania’ of State Affairs Department, National Representative of the European Association for Predictive, Preventive and Personalised Medicine (EPMA) in Ukraine, and a coordinator of the regional EPMA-BOARD in Ukraine; http://www.rostbubnov.narod.ru/.
